# Low-level laser therapy and conventional treatment for diabetic foot ulcers: a case series

**DOI:** 10.1007/s10103-026-04968-4

**Published:** 2026-07-31

**Authors:** Ariane Silva Pires, André Luiz Bandeira Dionizio Cardoso, Maysa Rodrigues Guedes, Francisco Gleidson de Azevedo-Gonçalves, Carolina Cabral Pereira da Costa, Patricia Ferracioli Siqueira Lemos, Hisadora Vaz de Souza, Bruna Maiara Ferreira Barreto Pires, Eugenio Fuentes Pérez Júnior

**Affiliations:** 1 Universidade do Estado do Rio de Janeiro, Rio de Janeiro, Brazil; 2 Instituto Estadual de Hematologia Arthur de Siqueira Cavalcanti, Rio de Janeiro, Brazil

**Keywords:** Laser therapy, Wound healing, Diabetes mellitus, Nursing care, Diabetic foot

## Abstract

Diabetic foot ulcers (DFUs) are a serious complication of diabetes mellitus and are associated with prolonged healing, increased hospitalization, and risk of amputation. Low-level laser therapy (LLLT), a non-invasive and low-cost modality, has shown potential to accelerate wound healing by promoting tissue repair, cellular proliferation, and improved microcirculation. This study aimed to descriptively evaluate healing outcomes in patients with DFUs treated with LLT combined with conventional treatment care with conventional treatment alone. This prospective observational comparative study included 11 adults with diabetes and active neuropathic DFUs treated at a public podiatry outpatient clinic in Rio de Janeiro, Brazil (June 2023–July 2024). Participants were prospective screened and allocated to conventional treatment (CT, *n* = 5; 6 ulcers) or LLLT plus CT (*n* = 6; 9 ulcers). Healing progression was assessed using the Pressure Ulcer Scale for Healing (PUSH), evaluating wound area, tissue type, and exudate. In the CT group, 1 ulcer (17%) healed completely, 2 (33%) improved, 2 (33%) remained unchanged, and 1 (17%) worsened. In the LLLT group, 5 ulcers (55.5%) healed completely and 4 (44.5%) improved, with no lesions worsening. The LLLT group also demonstrated shorter mean healing time, fewer clinical visits, and greater tissue repair than CT alone. The descriptive findings suggest that adjunctive LLLT may represent a promising supportive strategy for DFU management in real world outpatient care. Due to the observational design, non-random allocation, and limited sample size, causal inference cannot be established. Future randomized controlled trials are needed to confirm these findings.

## Introduction

Diabetes mellitus (DM) is a chronic and complex disorder characterized by impaired metabolism of glucose and other energy-producing substrates such as carbohydrates, fats, and proteins. This impairment results from a failure in insulin secretion or action, leading to a state of hyperglycemia [1]. Type 2 diabetes mellitus (T2DM) is the most prevalent type among adults and is associated with being overweight, physical inactivity, smoking and genetic factors [2].

Among its complications, diabetic foot disease is the most common, resulting from neuropathy, ischemia, or neuroischemia [3, 4]. Diabetic foot disease encompasses a range of pathophysiological processes arising from neurological abnormalities and/or vascular impairment, culminating in the development of ulcers with subsequent destruction of deep tissues, sometimes complicated by infections, and it is a major cause of limb amputation [3].

A neuropathic diabetic foot ulcer is characterized as a chronic, painless lesion surrounded by an area of hyperkeratosis, usually located on the plantar surface of the feet [4, 5]. In people with diabetes, impaired wound healing occurs due to chronic hyperglycemia, which causes alterations in the healing mechanism [6]. Granulation tissue in diabetic patients is poor in macrophages, exhibits reduced fibroblast proliferation, and shows impaired angiogenesis. Collagen stability is decreased due to glycation of collagen fibers, resulting in diminished wound contraction [7, 8].

In this context, the treatment of DFUs is based on principles that extend beyond local wound management and involve several measures, such as metabolic control, ulcer classification, assessment of location and depth, signs of infection, offloading and wound protection, local treatment of the wound bed and edges, as well as education of patients and their families. Vascular perfusion assessment is also emphasized for cases with vascular impairment or wounds that do not improve within 4–6 weeks [9, 10].

In recent years, adjunctive therapies have been tested to promote wound healing. Among these, low-level laser therapy (LLLT) has emerged as a non-invasive, painless, and low-cost method effective in wound treatment, acting on physiological and biochemical events in the healing process [10, 11]. LLLT emits low-power, non-ionizing electromagnetic radiation and acts as a biomodulator in cells and tissues, promoting tissue repair through effects such as cellular proliferation, tissue neoformation, revascularization, enhanced microcirculation, as well as anti-edematous and analgesic actions, making it an important tool in wound care [10–12].

Given the global burden of DM and its complications, particularly the high prevalence and lifetime risk of plantar ulcers, effective interventions are urgently needed [13]. Low-level laser therapy (LLLT) has emerged as a promising adjunct to conventional treatment, potentially accelerating wound healing and improving tissue repair. Therefore, this prospective observational comparative study aimed to descriptively evaluate healing outcomes associated with adjunctive LLLT combined with conventional care in patients with DFUs, focusing on wound closure progression, exudate reduction, and tissue repair.

## Methods

### Study design and setting

This prospective observational comparative study was conducted in the Podiatry Nursing Service of a public outpatient clinical complex in Rio de Janeiro, Brazil, from June 2023 to July 2024. This study was reported in accordance with Strengthening the Reporting of Observational Studies in Epidemiology (STROBE) recommendations [14].

### Participants

Participants with type 2 diabetes mellitus (T2DM) treated for neuropathic diabetic foot ulcers were prospectively identified through screening of active outpatient electronic clinical records. The sample included two groups based on treatment received: conventional treatment (CT, *n* = 5 patients with 6 ulcers) and conventional treatment combined with low-level laser therapy (LLLT, *n* = 6 patients with 9 ulcers).

Allocation to treatment groups was non-random and based exclusively on logistical feasibility for weekly outpatient attendance. Patients able to attend the clinic with the frequency required for weekly LLLT sessions were allocated to the adjunctive LLLT group. Patients unable to maintain this attendance schedule due to transportation or access-related constraints received conventional treatment alone.

### Eligibility criteria

Eligible records contain specific data on wound area, predominant tissue type, and exudate type necessary for the Pressure Ulcer Scale for Healing (PUSH) evaluation [15]. Exclusion criteria included osteomyelitis; antibiotic use during lesion treatment; inability to perform self-care due to cognitive or physical impairment; pregnancy; incomplete records; or treatment interruption during data collection.

### Ulcer classification

Diabetic foot ulcers were clinically classified according to the University of Texas Diabetic Foot Ulcer Classification System, routinely adopted in the study setting [16]. The PUSH scale was used exclusively for longitudinal monitoring of healing progression and not for baseline severity classification [15].

### Intervention

All patients shared the following treatment characteristics: weekly clinical follow-up and standardized wound care performed by nursing staff. This included wound cleaning with 0.9% saline solution, application of a primary calcium sodium alginate plate dressing, coverage with sterile gauze and crepe bandage, and occlusion with a bandage. All patients used removable offloading devices extending to the ankle.

Patients were instructed on home wound care, including protecting the wound during bathing with impermeable plastic film, performing hand hygiene, wearing gloves, wound cleaning with 0.9% saline solution, application of the primary alginate dressing, followed by secondary sterile gauze and occlusion with crepe bandage.

The LLLT group received low-level laser therapy once weekly, during dressing changes and after wound cleaning. The protocol consisted of combined red (660 nm) and infrared (808 nm) laser light, with a dose of 4 joules, applied point-by-point to 1 cm² areas (Table 1).

**Table 1:** Clinical characteristics of participants (*n* = 11) and treated foot ulcers (n =)

Patient	Group	Sex	Age	No. of ulcers	Ulcer location	PUSH basal	Medida inicial cm²	Class Texas	Hb1AC%
N1	CT	Male	68	1	Midfoot	3	0,9	1 A	7%
N2	CT	Female	49	2	Head of the 1 st and 5th metatarsals	5 and 7	2,2and 4,3	1 A	7,3%
N3	CT	Male	57	1	Hallux	10	24,5	1 A	7,6%
N4	CT	Male	65	1	Head of the 1 st metatarsal	8	9	1 A	6,8%
N5	CT	Female	60	1	Head of the 3rd metatarsal	6	3,5	1 A	7,1%
N6	LLLT	Male	62	2	Hallux and head of the 1 st metatarsal	3 and 3	0,8 and 0,9	1 A	6,9%
N7	LLLT	Male	67	1	Head of the 1 st metatarsal	7	4,3	1 A	7,5%
N8	LLLT	Female	59	1	Midfoot	6	3,2	1 A	7,9%
N9	LLLT	Male	65	2	Head of the 3rd and 5th metatarsals	6 and 3	3,4 and 0,8	1 A	7,5%
N10	LLLT	Female	63	1	Calcaneus	8	8,2	1 A	8,3%
N11	LLLT	Male	66	2	Head of the 1 st and 2nd metatarsals	4 and 3	1,2 and 0,9	1 A	6,9%

*CT* conventional treatment, *LLLT* low-level laser therapy, *No.* number

### Laser equipment specifications

Low-level laser therapy was applied once weekly immediately after wound cleansing and before dressing application. The protocol consisted of simultaneous red (660 nm) and infrared (808 nm) wavelengths delivered by the Easy Laser device [17].

The Easy Laser device (patented by Instituto Ricardo Trajano in 2009 and registered with ANVISA under number 80030819009) was used. It emits simultaneous low-power red and infrared laser beams (100 mW), in continuous mode via optical fiber, producing a collimated, highly directional light beam optimized for tissue penetration and wound healing stimulation.

Laser irradiation was applied in contact mode with the probe positioned perpendicular (90°) to the wound bed using a point-by-point technique over 1 cm² treatment areas (Fig. 3). A dose of 4 J was delivered per point. The device delivered 1 J every 10 s, resulting in 40 s of irradiation per 1 cm² treatment area [17]. Total session duration varied according to ulcer size.

### Data collection and outcome measures

Data from electronic medical records were used to complete the PUSH [15], used as a practical longitudinal wound-monitoring tool. Although originally developed for pressure ulcers, PUSH was adopted due to its operational applicability for serial wound assessment in routine clinical practice. PUSH evaluates wound healing based on three parameters: wound area (cm²), exudate amount (none, small, moderate, large), and wound bed appearance (predominant tissue type). The total score ranges from 17 (worst) to 0 (fully healed).

#### Data analysis

Data were descriptively summarized using Microsoft Excel^®^ 2024. The primary descriptive unit of analysis was the individual ulcer. Because some participants presented more than one lesion, observations were not statistically independent. No inferential statistical analyses were performed due to the limited sample size, clustering of ulcers within patients, and observational design. Results are presented descriptively.

### Ethical considerations

This study complied with ethical standards outlined in Resolution No. 466/2012 by the Brazilian National Health Council (CNS/MS) and was approved by the Research Ethics Committee under CAAE number 44704421.0.0000.5282, opinion 4.631.798.

## Results

### Clinical characteristics

A total of 11 patients (64% male, 36% female) with a mean age of 61 years were included, presenting 15 ulcers. Clinical and demographic characteristics of participants and lesions are summarized in Table 1. Regarding comorbidities, cardiovascular diseases were the most prevalent (81.8%), particularly systemic arterial hypertension (72.7%), followed by metabolic and endocrine disorders (54.5%), with dyslipidemia as the most frequent (45.4%) (Table 2). As for diabetes-related complications, diabetic neuropathy (47.2%), gait and bone alterations (20.9% and 24.9%, respectively) were the most common (Table 3). In addition, capillary blood glucose levels ranged from 108 to 211 mg/dL across consultations, with a mean of 196 mg/dL in both groups.

**Table 2:** Categories of associated comorbidities in participants

Category	Associated comorbidities
1	Cardiovascular diseases
2	Endocrine-metabolic disorders
3	Ophthalmologic diseases
4	Hepatic diseases
5	Psychiatric disorders
6	Osteoarticular disorders
7	Urological diseases
8	Malignant neoplasms
9	Gastrointestinal disorders
10	Neurological diseases
11	Respiratory diseases
12	Dermatological diseases
13	Infectious and parasitic diseases
14	Hematological disorders
15	Autoimmune diseases
16	Genetic syndromes

**Table 3:** Categories of diabetes-related complications in participants

Category	Diabetes-related complications
1	Neuropathy
2	Gait disturbance
3	Bone alterations
4	Peripheral arterial disease
5	Nephropathy
6	Diabetes-related foot ulcer
7	Amputations
8	Retinopathy
9	Charcot neuroarthropathy
10	Prosthesis use

### Conventional treatment

The CT group consisted of five patients and six treated ulcers (four patients presented with one ulcer each, and one patient presented with two ulcers). To systematize the results, each ulcer was designated with the letter “L” followed by an ordinal number from 1 to 6. The mean treatment duration in CT was 126 days, with an average of 12 clinical visits.

**Fig. 1:**
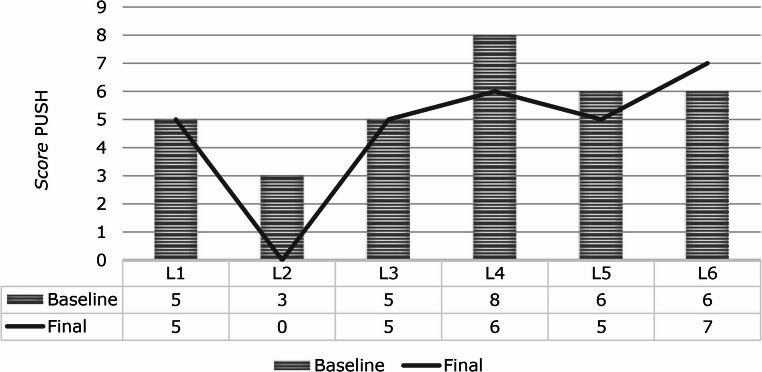
Baseline and final *scores* of the conventional treatment without LLLT, according to the PUSH scale

According to the PUSH scale, among the six analyzed ulcers (Fig. 1), only one (L2) achieved complete healing, while two others showed improvement as evidenced by reduced scores (L4 and L5). Two ulcers (L1 and L3) showed no changes in healing status, maintaining the same initial and final scores, and one ulcer (L6) worsened, with an increased score compared to baseline.

Regarding wound area (cm²), the following changes were observed: L1 decreased from score 3 (0.7–1.0 cm²) to 2 (0.3–0.6 cm²); L2 from 5 (2.1–3.0 cm²) to 0 (healed); L3 remained at 7 (4.1–8.0 cm²); L4 reduced from 10 (> 24 cm²) to 8 (8.1–12 cm²); L5 from 8 (8.1–12 cm²) to 7 (4.1–8.0 cm²); and L6 worsened, increasing from 6 (3.1–4.0 cm²) to 9 (12.1–24 cm²).

As for the amount of exudate, the second parameter assessed by the PUSH scale, three ulcers showed improvement: L4 and L5 decreased from score 2 (moderate amount) to 1 (small amount), and L2 from 1 to 0 (absence of exudate). Two ulcers maintained the same score of 1 (L3 and L6), and one ulcer (L1) worsened, increasing from 0 to 1.

For the last PUSH parameter, wound bed tissue type, the following results were obtained: four ulcers maintained their scores (L1, L3, L5, L6). Among them, L3 had a score of 3 (slough, a type of devitalized tissue), while L1, L5, and L6 scored 1 (epithelial tissue). One ulcer (L2) improved from 2 (granulation tissue) to 0 (healed), and another (L4) progressed from 2 (granulation tissue) to 1 (epithelial tissue).

### Low-level laser therapy

The LLLT group included six patients and nine ulcers (three patients had one ulcer each, and three patients had two ulcers). The mean treatment duration was 53 days, with an average of seven weekly laser therapy sessions per ulcer.

**Fig. 2:**
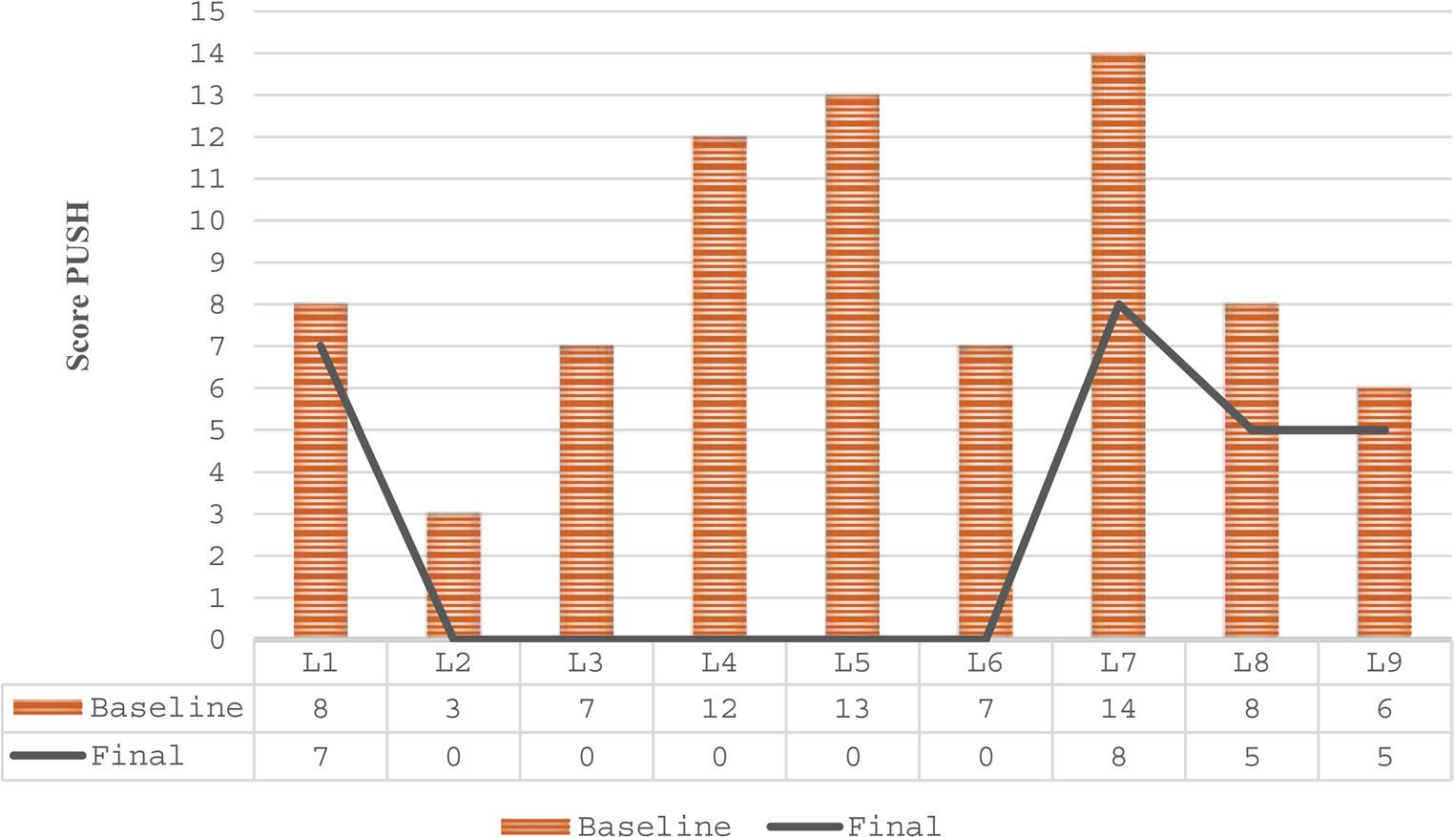
Baseline and final scores of the treatment with LLLT, according to the PUSH scale

According to the PUSH scale, five ulcers (L2, L3, L4, L5, L6) achieved complete healing, while the remaining four ulcers (L1, L7, L8, L9) improved with reduced scores compared to baseline (Fig. 2).

**Fig. 3:**
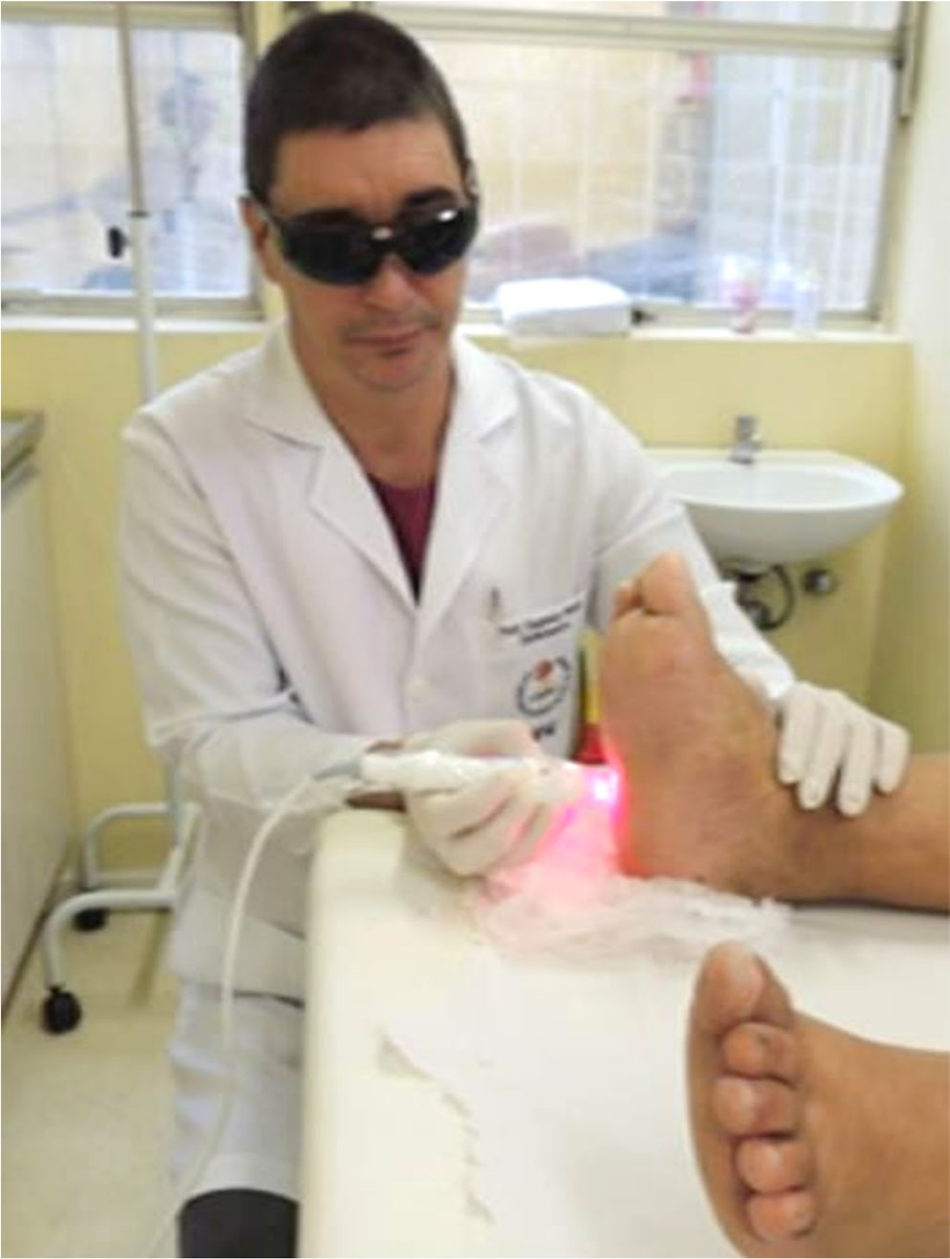
Application of low-level laser therapy (LLLT) to a diabetic foot ulcer (DFU), showing perpendicular (90°) probe positioning over the wound bed during treatment

Regarding wound area (cm²), according to the PUSH scale, five of the nine lesions achieved complete wound closure (score 0) at the end of treatment. Among these five fully healed lesions: L2 decreased from score 1 (< 0.3 cm²) to 0 (0 cm²); L3 from 7 (4.1–8 cm²) to 0; L4 from 6 (3.1–4.0 cm²) to 0; L5 from 8 (8.1–12 cm²) to 0; and L6 from 3 (0.7–1.0 cm²) to 0. The remaining four lesions (44.5%) showed partial improvement: L1 and L9 decreased from score 3 (0.7–1.0 cm²) to 2 (0.3–0.6 cm²); L7 from 8 (8.1–12 cm²) to 5 (2.1–3.0 cm²); and L8 from 4 (1.1–2.0 cm²) to 2 (0.3–0.6 cm²). Overall, all lesions demonstrated improvement in tissue repair (Fig. 3).

Regarding exudate amount, the second PUSH variable, two lesions remained unchanged (L1 score 2 [moderate] and L9 score 1 [small]), two lesions (L3 and L4) improved from score 3 (large) to 0 (absent), two lesions (L5 and L6) decreased from score 2 (moderate) to 0, two lesions (L7 and L8) improved from 2 to 1, and one lesion (L2) improved from 1 to 0.

Concerning the wound bed tissue type, the final PUSH variable, the five fully healed lesions showed the following changes: three lesions (L3, L4, L5) improved from score 3 (slough) to 0 (healed), L2 improved from 1 (epithelial tissue) to 0, and L6 from 2 (granulation tissue) to 0. Of the four lesions that were partially improved, three lesions maintained the same tissue type from baseline to the end of treatment (L8 and L9: score 2 [granulation]; L1: score 3 [slough]), and one lesion (L7) improved from score 4 (necrotic tissue) to 2 (granulation).

Analyzing outcomes across groups, in the conventional treatment group, 1 lesion (17%) healed completely, 2 lesions (33%) improved, 2 lesions (33%) remained unchanged, and 1 lesion (17%) worsened. In the LLLT group, 5 lesions (55.5%) healed completely, and 4 lesions (45.5%) improved partially, resulting in a healing prevalence of 55.5%.

Furthermore, despite a higher number of lesions with larger wound areas in the LLLT group, the mean healing time and the number of sessions per lesion were considerably lower than in the conventional treatment group. Notably, no lesions treated with LLLT showed worsening of the healing process.

Descriptively, complete healing was observed more frequently among ulcers managed with adjunctive LLLT, whereas no lesion worsening was observed in this group during follow-up.

## Discussion

DFUs are a common complication in patients with diabetes, often leading to delayed wound healing, infection, and increased risk of amputation [18]. Adjunctive therapies such as LLLT have been investigated for their potential to accelerate tissue repair and improve clinical outcomes. In this context, the current case series aimed to evaluate the effects of LLLT combined with conventional treatment in patients with DFUs, assessing wound closure, exudate reduction, and tissue repair.

According to the International Diabetes Federation, DFUs represent a significant clinical challenge, reflecting the vulnerability of lower limbs in patients with diabetes and the high risk of progression to severe complications, including amputation [16]. Several risk factors contribute to delayed healing in DFUs, such as neuropathy, peripheral arterial disease, previous ulceration, advanced age, and poor self-care practices [19, 20]. Patients who received LLLT in addition to conventional treatment exhibited accelerated wound closure, reduced exudate, and improved tissue repair compared to those receiving conventional care alone, suggesting that laser therapy may mitigate negative factors by enhancing cellular proliferation, extracellular matrix synthesis, and local microcirculation.

Among therapeutic modalities, LLLT has been recognized for its potential as a safe, noninvasive, and low-cost adjunctive intervention. Previous studies have shown physiological effects including anti-inflammatory action, tissue regeneration, modulation of cellular activity, and analgesia [21, 22]. The present study confirms these benefits in a real-world clinical setting, showing faster wound closure and improved exudate control in the LLLT-treated group, even when accounting for baseline ulcer characteristics.

Previous systematic review have suggested that photobiomodulation may contribute to improved healing outcomes in DFUs, particularly regarding wound area reduction and tissue repair acceleration [21]. In addition, a randomized clinical trial, showed that LLLT acts on cells and biochemical processes during the inflammatory phase of wound repair, enhancing cell proliferation and extracellular matrix synthesis during remodeling [23]. By acting as a biomodulator, LLLT promotes new tissue formation, improves blood circulation, reduces swelling, and provides pain relief [23]. Likewise, literature reviews emphasize that LLLT offers tissue regeneration, anti-inflammatory, analgesic, and healing properties [24–26]. Other systematic reviews have also shown that LLLT, even when not achieving complete closure of the lesion, can accelerate wound healing and improve patient well-being [26, 27].

In the present study, patients who received LLLT in combination with conventional treatment showed notable improvements in tissue repair, exudate reduction, and wound closure, consistent with evidence from previous investigations [28, 29]. In contrast, patients treated with conventional care alone also improved but demonstrated a slower and less pronounced healing response.

Supporting these findings, another study carried out in Caxias do Sul, Brazil, reported ulcer healing in 77% of patients treated with LLLT, compared with 66% among those treated conventionally after 16 weeks. Although no statistically significant differences were detected between groups, LLLT patients presented higher quality-of-life scores, particularly regarding mobility, as measured by the EuroQol-5D instrument—indicating an additional clinical benefit beyond wound healing [30].

Taken together, these findings suggest that LLLT, when combined with conventional treatment, may enhance healing outcomes and improve patient quality of life, reinforcing its role as a promising adjunctive therapy. From a nursing perspective, the use of validated and evidence-based tools such as LLLT highlights the potential for expanding professional practice and improving patient care.

The findings of this study should be interpreted considering several important limitations. The small sample size and single-center design restrict external validity. Non-random allocation based on logistical feasibility introduces potential selection bias, since participants able to attend weekly sessions may differ in adherence-related characteristics. Additionally, multiple ulcers occurring in the same patient may share systemic influences, limiting lesion-level independence. The use of PUSH, although operationally practical, represents a limitation because it was originally validated for pressure ulcers rather than diabetic foot ulcers specifically. The absence of inferential statistical analysis precludes comparative effectiveness conclusions. These preliminary descriptive observations should therefore be interpreted cautiously.

## Conclusion

This prospective observational comparative study descriptively identified favorable healing patterns among diabetic foot ulcers managed with adjunctive LLLT combined with conventional treatment. Although these findings support the clinical feasibility of LLLT as a supportive therapeutic strategy, methodological limitations prevent causal interpretation. Future randomized controlled multicenter studies with standardized dosimetry and larger samples are necessary to determine clinical effectiveness and optimize treatment protocols.

## Data Availability

No datasets were generated or analysed during the current study.
